# Gold Nanoparticle-Bioconjugated Aminoguanidine Inhibits Glycation Reaction: An *In Vivo* Study in a Diabetic Animal Model

**DOI:** 10.1155/2021/5591851

**Published:** 2021-05-13

**Authors:** Saheem Ahmad, Mohd. Sajid Khan, Sultan Alouffi, Saif Khan, Mahvish Khan, Rihab Akashah, Mohammad Faisal, Uzma Shahab

**Affiliations:** ^1^Department of Clinical Laboratory Sciences, College of Applied Medical Sciences, University of Hail, Saudi Arabia; ^2^Department of Biosciences, Integral University, Lucknow, India; ^3^Department of Biochemistry, Faculty of Life Sciences, Aligarh Muslim University, Aligarh 202002, India; ^4^Molecular Diagnostic & Personalized Therapeutic Unit, University of Hail, Saudi Arabia; ^5^Department of Basic Dental and Medical Sciences, College of Dentistry, University of Hail, Ha'il 2440, Saudi Arabia; ^6^Department of Biology, College of Science, University of Hail, Ha'il 2440, Saudi Arabia; ^7^Department of Botany and Microbiology, College of Science, King Saud University, Saudi Arabia; ^8^Department of Biotechnology, Khwaja Moinuddin Chishti Language University, Sitapur-Hardoi Bypass Road, Lucknow 226013, India

## Abstract

Proteins undergo glycation resulting in the generation of advanced glycation end products (AGEs) that play a central role in the onset and advancement of diabetes-associated secondary complications. Aminoguanidine (AG) acts as an antiglycating agent by inhibiting AGE generation by blocking reactive carbonyl species (RCS) like, methylglyoxal (MGO). Previous studies on antiglycating behavior of AG gave promising results in the treatment of diabetes-associated microvascular complications, but it was discontinued as it was found to be toxic at high concentrations (>10 mmol/L). The current article aims at glycation inhibition by conjugating gold nanoparticles (Gnp) with less concentration of AG (0.5-1.0 mmol/L). The HPLC results showed that AG-Gnp fairly hampers the formation of glycation adducts. Moreover, the *in vivo* studies revealed AG-Gnp mediated inhibition in the production of total-AGEs and -*N*^*ε*^-(carboxymethyl)lysine (CML) in the diabetic rat model. This inhibition was found to be directly correlated with the antioxidant parameters, blood glucose, insulin, and glycosylated hemoglobin levels. Furthermore, the histopathology of AG-Gnp-treated rats showed good recovery in the damaged pancreatic tissue as compared to diabetic rats. We propose that this approach might increase the efficacy of AG at relatively low concentrations to avoid toxicity and might facilitate to overcome the hazardous actions of antiglycating drugs.

## 1. Introduction

In diabetes mellitus, hyperglycemia-mediated glycation starts with the nucleophilic addition between the carbonyl group of reducing sugars with free amino groups of proteins [1–3]. The glycation reaction proceeds with the formation of reversible Schiff's bases, to stable ketoamines or Amadori products, which finally lead to the formation of advanced glycation end products (AGEs) [4, 5]. Moreover, autoxidation of glucose and glycoxidation of proteins give rise to reactive oxygen species (ROS) that lead to the generation of reactive carbonyl species (RCS) or dicarbonyls such as glyoxal (GO), methylglyoxal (MGO), and 3-deoxyglucosone (3-DG) [6, 21]. Furthermore, shunting of excess glucose in the polyol pathway and glycolysis too results in the generation of RCS [7–9].

In the physiological system, levels of reducing sugars and RCS together determine the type of AGEs that are generated during glycation reaction [10, 11]. The *in vivo* formation and accretion of AGEs such as -*N*^*ε*^-(carboxymethyl)lysine (CML), -*N*^*ε*^-(carboxyethyl)lysine (CEL), *ε*-fructosyl-L-lysine (FL), argpyrimidine, and pentosidine play a decisive role in the pathogenesis of diabetes-associated secondary complications and cancer [12, 13]. Although glycation is a slow process but increases several folds during persistent hyperglycemia [14], plasma amines react with the carbonyl group of sugars, Amadori products, and RCS, thereby preventing the nucleophilic addition reaction between >C=O and –NH_2_ [15]. Furthermore, antioxidants quench glycation-derived ROS, whereas transport proteins such as ceruloplasmin bind with the transition metal ions and prevent them to participate in autoxidation and glycoxidation reactions [16, 17].

Aminoguanidine (AG), a hydrazine compound, is an antiglycation drug that inhibits AGE generation via blocking the carbonyl groups on reducing sugars, Amadori products, and RCS [18, 19]. Earlier investigations on AG gave promising results in the treatment of diabetes-associated microvascular complications such as retinopathy and nephropathy but were later on found to be toxic at higher concentrations (>10 mM), and hence for safety concerns, the human clinical trials were halted [20]. However, several *in vitro* investigations and animal studies have shown AG-mediated prevention of diabetes-induced deterioration in diabetes-associated cardiovascular diseases [21].

In the light of the aforementioned accounts, this study is aimed at exploiting the preventive effects of AG on glycation and diabetes, by using gold nanoparticle- (Gnp-) bioconjugated AG (AG-Gnp) at low concentrations which is nontoxic. Gold nanoparticles (Gnp) are among the most commonly used nanostructures in biological applications [22, 23]. Gnp increase drug effectiveness due to the virtue of their biocompatibility, surface area, and surface functionalization and hence are frequently used in the cure of chronic lymphocytic leukemia [24, 25]. Gnp can easily conjugate with different globular proteins like BSA and cytochrome c [26]. Interestingly, Gnp has been utilized by several investigators in determining the glycation status of the proteins [27]. Furthermore, diabetic rat models were designed to check the efficacy of AG-Gnp *in vivo* by measuring certain markers of glycation including total AGEs and CML-AGEs, and antioxidant status of the diabetic and treated animals as well.

## 2. Materials and Methods

### 2.1. Materials

#### 2.1.1. Chemicals

Methylglyoxal (MGO), human serum albumin (HSA), aminoguanidine (AG), alloxan, and *α*-NADPH were obtained from Sigma-Aldrich. The lactate dehydrogenase (LDH) kit was purchased from Biomedical Research Services (Buffalo, NY, USA). 5,5′-Dithio-bis-[2-nitrobenzoic acid] (DTNB) was bought from Pierce Biotechnology (Rockford, IL). All other reagents and solvents were of the highest analytical grade which were obtained from HiMedia, Mumbai, India.

#### 2.1.2. Animals

The male Wistar rats (200–220 gm) were purchased from the Central Drug Research Institute, Lucknow, India. The animals were kept in a 12/12-hour light and dark cycle in temperature-controlled cages with free access to normal diet and water. The study was approved by “The Institutional Animal Ethics Committee” of Integral University, Lucknow, India, with approval number: IU/Biotech/Project/CPCSEA/13/12.

### 2.2. Methods

#### 2.2.1. *In Vitro* Synthesis of Gold Nanoparticles and Its Bioconjugation

Gold nanoparticles (Gnp) were prepared by the method described earlier with slight modifications [28]. The Gnp were conjugated with aminoguanidine by using 1-ethyl-3-(3-dimethylaminopropyl)-carbodiimide (EDC) [29, 30].

#### 2.2.2. Preparation of Glycated Samples

The glycated samples were prepared as described previously [31, 32]. In brief, 20 mg mL^−1^ HSA was incubated with 10 mmol/L methylglyoxal (MGO) in 20 mmol/L phosphate buffer saline (PBS) of pH 7.4. The reaction mixtures were kept at 37°C for 20 days under strict sterile conditions to avoid any microbial growth by adding 0.02% sodium azide. Blanks, HSA (20 mg mL^−1^), and MGO (10 mmol/L) were also incubated separately for the same time.

#### 2.2.3. Reverse Phase-High Performance Liquid Chromatography (RP-HPLC) Analysis

RP-HPLC analysis was performed on a Hitachi analytical HPLC system comprising of L-7100 low-pressure gradient pumps, L-7200 sequential autosampler, and a high sensitivity diode array detector (190–800 nm) and was managed by D-7000 HPLC system manager software. Reverse phase-HPLC contains a c-18 column for the separation of samples. Mobile phases A and B were 0.1 mol/L aqueous ammonium acetate and 100% HPLC analytical grade acetonitrile, respectively. A stepwise elution of 90 : 10 of B : A of the mobile phases was applied for 15 min followed by 10 : 90 of B : A of mobile phases for another 15 min in the entire analysis. There are 10% acetonitrile and 90% H_2_O with 0.1% HCOOH as solvent A and 90% acetonitrile and 10% H_2_O with 0.1% HCOOH as solvent B. Gradients started from 0% B to 60% B after 30 min, then 90% B after another 15 min and then again brought to 0% B. Before HPLC analysis, all the solvents were filtered with 0.45 *μ*m membranes (Millipore, USA) and degassed for 10 min before use. All the profiles were obtained at a fixed wavelength of 280 nm.

In another set of experiments of HPLC, determination of -*N*^*ε*^-(carboxymethyl)lysine (CML) by HPLC was done as described earlier [33]. In brief, 10 mL of 35% HCl was used to hydrolyze the 10 mg sample at 105°C. After hydrolysis, the sample was centrifuged at 14000 rpm for 15 minutes after cooling at room temperature. 10 mL of methanol and 20 mL of deionized water (DW) was used to prewet Sep-Pak C18 cartridge (Waters, MA, U.S.A.), and supernatant (1 mL) was applied to it and subsequently eluted with 3 M HCl (10 mL). The eluted sample (20 *μ*L) and o-phthalaldehyde (OPA) reagent (20 *μ*L) were mixed vigorously. After 3 minutes, derivatized hydrolysate (20 *μ*L) was analyzed at 32°C with an analytical column (4.6 × 250 mm). The mobile phase was composed of (A) DW and (B) methanol, and the gradient was linear from 0 to 100% B in 16 min. The OPA derivatives were excited at 340 nm, and the emission was recorded at 440 nm on an Agilent Cary Eclipse Fluorescence Spectrometer. The peak retention time of the CML standard (PolyPeptide Lab., CA, USA) was used to identify the peak of sample constituents.

#### 2.2.4. Lactate Dehydrogenase (LDH) Assay

Lactate dehydrogenase assay was done by using the LDH kit (Biomedical Research Services) as described earlier [34]. In brief, the blood was centrifuged at 400 rpm to obtain platelet-rich plasma. AG (10 mmol/L) and AG-Gnp (0.5 and 1.0 mmol/L) were incubated for 2 hr with platelets. The cytotoxicity of AG and AG-Gnp was measured by the release of LDH from platelets lysed with 1% Triton X-100.

#### 2.2.5. Alloxan-Induced Diabetes in Male Wistar Rats

Type 1 diabetes in Wistar rats was induced by giving an intraperitoneal single dose of alloxan (100 mg/kg) after 24 hr of fasting. The alloxan was dissolved in 0.9% NaCl. The animals were housed in a standard laboratory condition, 12-hour light-dark cycle, constant temperature, and easy access to food and water. The induction of hyperglycemia was confirmed one week after treatment. Rats with a blood glucose level of ≥11 mmol/L were considered diabetic [35].

#### 2.2.6. Measurement of AGE and CML Levels in Diabetic and AG-Gnp-Treated Groups

Total AGEs in the serum samples of experimental rats were measured by using the ELISA kit from Cell Biolabs (San Diego, CA). Anti-AGE polyclonal antibody and horseradish peroxidase-conjugated secondary antibody were used to probe AGEs, though the total AGE detection and inhibition in the serum samples are not precise by this method. However, we performed this study just to see the overall AGE inhibition as a result of AG-Gnp. The AGE content was determined by comparing with AGE-bovine serum albumin (BSA) standard curve ranging from 0.25 to 5 *μ*g mL^−1^. The absorbance was read at 450 nm on a Bio-Rad Multiplate Reader (Bio-Rad, Laboratories, CA) against BSA standard. AGE-BSA contains pentosidine, CML, and other heterogenous AGE structures.

Similarly, -*N*^*ε*^-(carboxymethyl)lysine in the serum samples was also measured by using the ELISA kit from Cell Biolabs (San Diego, CA). The CML contents were determined by comparing it with CML-BSA standard curve ranging from 0.035 to 2.2 ng mL^−1^. The rest of the procedure was the same as that of the total AGE estimation [36].

#### 2.2.7. Effect of AG and AG-Gnp on Blood Glucose, Triglycerides, and Insulin in a Diabetic Rat Model

Glucose levels were estimated to ensure induction of T1DM in experimental rats. Six rats were randomly selected as a normal group, and the rest were used to induce a diabetic model. AG (10 mM/kg body weight) was used as control and administered intraperitoneally for 2 weeks on daily basis. The concentration of AG-Gnp was 0.5 mmol/L and 1.0 mmol/L per kg body weight. The AG and AG-Gnp treatment was further maintained by including the drugs in drinking water for two more weeks. After four weeks of AG and AG-Gnp administration, blood was drawn from the oculo-orbital vein after 12 hr fasting and the levels of insulin, glucose, and triglycerides were determined [37, 38].

#### 2.2.8. Effect of AG and AG-Gnp on the Antioxidant Defense System and Pancreatic Exocrine Function in Diabetic Rats

The diabetic rats were arbitrarily segregated into four groups (*n* = 8 in each group): (a) diabetic, (b) diabetic+AG (10 mM/kg), (c) diabetic+AG-Gnp (0.5 mM/kg), and (d) diabetic+AG-Gnp (1.0 mM/kg), respectively. After four weeks of drug administration, animals fainted with ethyl carbamate (1.4 g/kg, i.p.) and the collected blood was centrifuged at 3000 rpm for 5 min. The concentrations of malondialdehyde (MDA), reduced glutathione (GSH), and the activities of superoxide dismutase (SOD), catalase (CAT), and amylase (AMS) in the sera were measured. After blood collection, the rats were sacrificed by decapitation and the pancreatic tissues were removed for histological investigations.

#### 2.2.9. Histopathology of Diabetic and AG- and AG-Gnp-Treated Rat Pancreas

The effect of diabetes on animal tissue was examined by histopathology of rat pancreas as described previously [39]. In brief, pancreatic tissue fragments were fixed in 10% formalin solution, embedded in paraffin, and stained with hematoxylin and eosin. The tissue slides were assessed by bright-field microscopy (Carl Zeiss, Axiolab 5, Germany).

#### 2.2.10. Statistical Analysis

The results were evaluated by using analysis of variance (ANOVA) followed by Newman-Keuls multiple comparisons. In general, the null hypothesis used for all analyses was that the factor does not influence the measured variables, and significance was accepted at the over 95% confidence level.

## 3. Results

### 3.1. Absorption Profile of Aminoguanidine-Bioconjugated Gold Nanoparticles

The absorption spectrum of aminoguanidine gold nanoparticles (Gnp-HSA-AG) showed a peak at 529 nm that corresponds to AG-gold nanoparticles (AG-Gnp) with a blue shift suggesting its binding to Gnp.

### 3.2. Cytotoxicity Assay

Cytotoxicity assay showed no significant increase in LDH leakage after incubation of platelets with 0.5 and 1.0 mmol/L AG-Gnp. With 0.5 mmol/L AG-Gnp, the release of LDH was 3.91 ± 0.56%, whereas, with 1.0 mmol/L concentration of AG-Gnp, it was 4.13 ± 0.48%. However, at 10 mmol/L concentration of AG, the increase in LDH was found to be quite high, i.e., 7.13 ± 1.10%. It is important to note that more than 4 ± 0.51% of LDH leakage is supposed to be toxic at this concentration. The results are summarized in Table 1.

### 3.3. RP-HPLC Analysis

**Figure 1:**
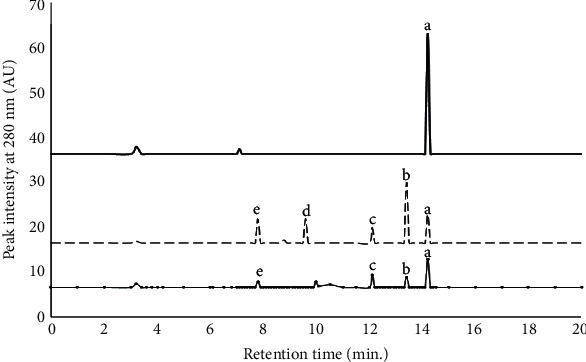
HPLC elution profiles of HSA with MGO mixtures after 15 days of incubation; HSA (─), HSA-MGO (-----), and HSA-MGO with 1.0 mM AG-Gnp (─•─•─). UV absorbance was measured at 280 nm wavelength. All readings were taken in triplicates.

The samples to be analyzed were hydrolyzed with 6 N HCl for 24 hr at 110°C. The hydrolyzed samples were then filtered by a 0.42 *μ*M Millex filter for ultrafiltration. The HPLC elution profiles of the hydrolysated HSA, HSA-MGO, and HSA-MGO with AG-Gnp (1.0 mmol/L) are shown in Figure 1. HSA was eluted alone at 14.2 minutes (peak a), whereas MGO did not show a separate peak at 280 nm. Upon glycation, i.e., in HSA-MGO, a sequence of newly emerged peaks (b, c, d, and e) represents the formation of AGEs, whereas peak “a” that represents unmodified HSA decreased simultaneously. This indicates that HSA has lost its native state upon glycation with MGO. The additional peaks b, c, d, and e in HSA-MGO were eluted at the retention time of 13.4, 12.1, 9.6, and 7.8 minutes, respectively.

The elution profile of HSA-MGO with AG-Gnp (1.0 mmol/L) showed only 3 peaks (b, c, and e) with decreased intensities that correspond to AGEs. Peak “d” was absent in the presence of AG-Gnp which was one of the AGEs formed in MGO-glycated HSA (HSA-MGO). The decreased intensities for peaks b, c, and e matched to 84.2 ± 4.2%, 68.7 ± 5.4%, and 70 ± 3.5%, respectively. These results confirmed that fewer AGE species were formed in the presence of AG-Gnp at 1.0 mmol/L concentration.

**Figure 2:**
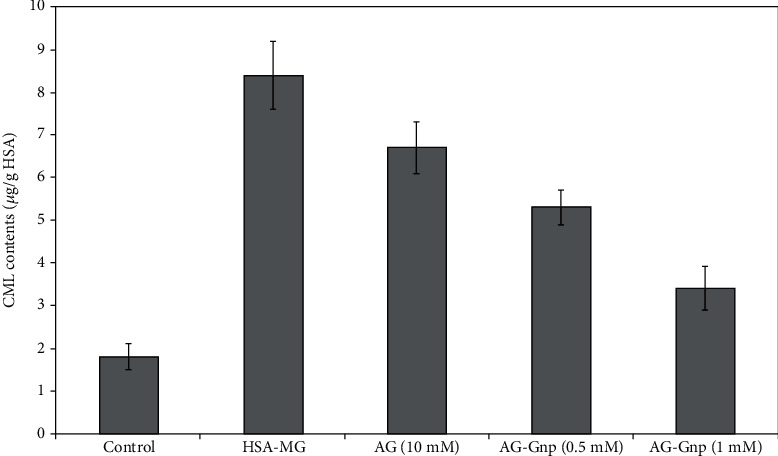
CML contents (n gm^−1^) in the reaction mixture of MGO-HSA mixtures. The inhibitions of the CML contents were measured using 10 mM AG, 0.5 mM, and 1.0 mM AG-Gnp. All experiments were performed in triplicates.

The antiglycation effect of AG-Gnp on the level of representative AGE marker, i.e., carboxymethyl lysine (CML) is shown in Figure 2. The AG treatment significantly inhibited (34.5 ± 1.8%) the formation of CML to nontreated glycation control (HSA-MGO). When treatment was done with 0.5 mmol/L AG-Gnp, the inhibition was as higher as 48 ± 2.6% in comparison to AG, whereas, with 1.0 mmol/L AG-Gnp, the CML inhibition was found to be 72.1 ± 3.2%.

### 3.4. Effects of AG and AG-Gnp on Blood Glucose, Triglycerides, and Insulin Profile in a Diabetic Rat Model

The alloxan-mediated damage suffered by pancreatic *β*-cells resulted in high blood glucose and triglyceride levels and reduced insulin levels in diabetic groups with respect to normal control groups. The administration of AG (10 mM/kg) and AG-Gnp (0.5 and 1.0 mM/kg) reduced the blood glucose and triglyceride levels and increased the insulin levels. The AG-Gnp- (0.5 and 1.0 mmol/L) treated group of animals was higher in inhibiting the levels of blood glucose and triglycerides and enhancing the level of insulin than the AG group. The AG-Gnp group was able to rectify the above parameters in a dose-dependent manner. The 1.0 mM/kg group of AG-Gnp was found to be more pronounced than the 0.5 mM/kg group of AG-Gnp, in reducing the levels of triglycerides and blood glucose and enhancing the level of insulin. Compared with diabetic or AG groups, AG-Gnp at 1.0 mM/kg groups exhibited a twofold increase in insulin levels, which suggests that AG-Gnp might exert some protective effect on the pancreatic tissues or *β*-cell function. The results are summarized in Table 2.

### 3.5. Effects of AG and AG-Gnp on Antioxidant Activity and Pancreatic Exocrine Function in a Diabetic Rat Model

Malondialdehyde (MDA) equivalent concentration in the diabetic group (4.23 ± 0.89 nmol/L) was higher than that in the control group (2.45 ± 0.68 nmol/L), which reflects increased lipid peroxidation. However, with respect to the diabetic group, all the treated groups showed a reduction in MDA levels, i.e., AG (10 mmol/L) = 3.27 ± 0.98, AG-Gnp (0.5 mmol/L) = 3.13 ± 0.43, and AG-Gnp (1.0 mmol/L) = 2.87 ± 0.42 nmol/L, which indicates reduced lipid peroxidation. The superoxide dismutase (SOD) activity in the diabetic group (201.9 ± 21.7 U/mL) was much higher than that in the normal group (140.2 ± 12.2 U/mL). However, with respect to the diabetic group, all the treated groups showed a reduction in SOD activity, i.e., AG (10 mmol/L) has 136.3 ± 56.4, AG-Gnp (0.5 mmol/L) recorded to be 130.2 ± 43.6, and AG-Gnp (1.0 mmol/L) found to be 136.6 ± 25.2 U/mL. The catalase (CAT) activity in the diabetic group (1.48 ± 0.83 U/mL) was much lower than that in the normal group (4.22 ± 1.87 U/mL). However, with respect to the diabetic group, all the treated groups showed an increase in CAT activity, i.e., AG (10 mmol/L) = 3.21 ± 1.14, AG-Gnp (0.5 mmol/L) = 3.9 ± 1.45, and AG-Gnp (1.0 mmol/L) = 4.1 ± 1.11 U mL^−1^. The glutathione (GSH) activity in the diabetic group (123.4 ± 12.5 mg dL^−1^) was lower than that in the normal group (172.9 ± 13.5 mg dL^−1^). However, with respect to the diabetic group, all the treated groups showed an increase in GSH activity, i.e., AG (10 mmol/L) = 155.8 ± 42.3, AG-Gnp (0.5 mmol/L) = 167.8 ± 34.6, and AG-Gnp (1.0 mmol/L) = 178.1 ± 34 mg dL^−1^. The pancreatic amylase (AMS) activity in the diabetic group (617.3 ± 58.4 U/dL) was lower than that in the normal group (719.8 ± 23.7 U/dL). However, in comparison to the diabetic group, all the treated groups showed an increase in AMS activity, i.e., AG (10 mmol/L) = 630.4 ± 93.5, AG-Gnp (0.5 mmol/L) = 670.8 ± 72.6, and AG-Gnp (1.0 mmol/L) = 701.4 ± 68.5 U/dL. The results are summarized in Table 3.

### 3.6. Determination of Total AGE and CML Contents

The AGE levels in the sera of normal control and diabetic rats were found to be 1.21 ± 0.23 and 5.83 ± 0.45 *μ*g mL^−1^, respectively. Upon treatment with 10 mmol/L AG, the levels of AGEs were 4.92 ± 0.63 *μ*g mL^−1^. However, the concentration of serum AGEs in the case of 0.5 and 1.0 mmol/L of AG-Gnp-treated diabetic rats showed 4.14 ± 0.53 and 3.21 ± 0.71 *μ*g mL^−1^, respectively. The decline in the level of the serum AGEs in the case of 0.5 mmol/L AG-Gnp was recorded to be 28.8 ± 4.2%, and when the concentration of AG-Gnp was further increased to 1.0 mmol/L, the level of AGEs was further reduced to 44.9 ± 5.7%.

The CML is among the best-studied AGEs in the context of diabetes and its secondary complications. The CML levels in the sera of normal control and diabetic rats were 0.52 ± 0.16 and 2.12 ± 0.84 ng mL^−1^, respectively. Upon treatment with 10 mmol/L AG, the levels of CML were 2.01 ± 0.23 ng mL^−1^, which is 5.1 ± 0.70% less than the diabetic rats. However, the concentration of serum CML in the case of 0.5 and 1.0 mmol/L of AG-Gnp-treated diabetic rats showed 1.68 ± 0.33 and 1.23 ± 0.18 ng mL^−1^, respectively. The drop in the level of the serum CML in the case of 0.5 mmol/L AG-Gnp was recorded to be 20 ± 1.6%, and when the concentration of AG-Gnp was further increased to 1.0 mmol/L, the level of CML was further reduced to 41 ± 0.71%. The results are summarized in Table 4.

### 3.7. Histopathology of Rat Pancreas

**Figure 3:**
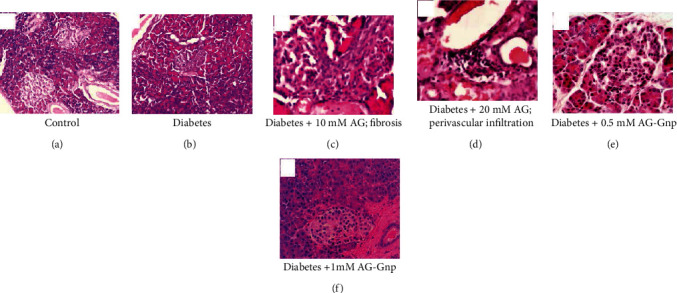
Effects of 10 and 20 mM of AG and 0.5 and 1 mM of AG-Gnp on pancreatic islet tissues in alloxan-induced diabetic rats. Photomicrographs showing pancreatic islet of the (a) normal control group, (b) diabetic group, (c) diabetic+10 mM/kg AG group, (d) diabetic+20 mM/kg AG group, (e) diabetic+0.5 mM AG-Gnp, and (f) diabetic+0.5 mM AG-Gnp. All experimental groups contain 6-8 rats.

The pancreatic tissues were typical in the control group with normal exocrine glands and islets of Langerhans (Figure 3(a)). However, in diabetic rats, the pancreatic tissues stained with H&E were found to be degenerative and necrotic along with shrinkage in Langerhans cells (Figure 3(b)). When diabetic rats were treated with 10 mM AG, the occurrence of cellular fibrosis suggested the prooxidant activity of AG (Figure 3(c)). However, when AG concentration was increased to 20 mmol/L, a pathetic situation called perivascular infiltration of lymphocytes occurred (Figure 3(d)). These results are in agreement with the LDH assay, where we found cytotoxicity of AG at 10 mmol/L concentration. However, when 0.5 mmol/L AG-Gnp was given to diabetic rats, the disrupted islet boundaries and mass distribution of cytoplasm were improved, implying replenishment of islet structure and cell addition in the islets of diabetic rats (Figure 3(e)). Moreover, the administration of 1 mM AG-Gnp markedly repaired this islet damage and improved the condition of islet cells and tissues (Figure 3(f)).

## 4. Discussion

The glycation-mediated AGE generation affects human physiology and is responsible for the pathogenesis of diabetes-associated secondary complications [40–42]. In this study, we evaluate the effect of gold nanoparticles (Gnp) on AGE generation during methylglyoxal- (MGO-) mediated glycation of human serum albumin (HSA) *in vitro* and diabetic rat model. Earlier, it has been studied that protein amino groups and gold colloids both interact with each other [43]. Although methodological studies demonstrated that larger nanoparticles could aggregate, it has not been reported that Gnp tends to aggregate while reacting with proteins [44]. Nanoparticles have a very large surface-to-volume ratio so that even a small amount of particles present extremely large surface areas available for protein binding [45]. Aggarwal et al. reported that proteins such as albumin, immunoglobulins, and fibrinogen associate with a wide range of nanoparticles of varying size and produce diverse molecular compositions [46]. As per previous reports, there are a variety of AGEs present in the circulatory system like CML, *N*^*ε*^-(carboxymethyl)arginine, and -*N*^*ε*^-(carboxyethyl)lysine [47]. Our results illustrated that the reactivity between MGO and HSA and the destabilization of HSA by MGO were decreased by AG-Gnp in a concentration-dependent manner. We hypothesized that AG-Gnp might compete with common glycation sites of HSA and obstruct the amino groups from reacting with MGO. A few of the free amino groups of protein can react with Gnp by donating electrons to the AG-Gnp, and AG-Gnp also can form electrostatic interactions with protonated amino groups. The HPLC data confirmed that the formation of AGEs shrank in the presence of AG-Gnp in a concentration-dependent manner. The drop in AGE levels might be due to the inhibition of MGO-mediated glycation of HSA by AG-Gnp, although AG (10 mmol/L) too moderately inhibited the formation of end-stage glycation adducts but to a lesser extent than AG-Gnp. However, the LDH assay discourages the use of AG because of its cytotoxic effect.

No matter the cause of diabetes, the result is always hyperglycemia. Studies have revealed hyperglycemia-mediated pathogenesis of microvascular complications such as neuropathy, nephropathy, and retinopathy. Various mechanisms have been proposed to explain the increased vascular risk associated with hyperglycemia. One such mechanism is the augmented formation of AGEs by the protein glycation. Clinically, type 1 diabetes is characterized by a typical autoimmune assault against the *β*-cells, inducing progressive *β*-cell death which is in line with the progressive decline in first-phase insulin secretion and causes insulin insufficiency and hyperglycemia [48].

In the alloxan-induced type 1 diabetic model, a significant increase in insulin and reduction in glucose levels were reported after AG-Gnp administration, along with an improved lipid profile. These results indicate the efficiency of AG-Gnp treatment in comparison to AG alone. Moreover, the results also implied AG-Gnp mediated protecting effect on pancreatic tissues and *β*-cell functioning, which were further validated by investigating the activity of pancreatic amylase (AMS) and the antioxidant defense system such as superoxide dismutase (SOD), catalase (CAT), and glutathione (GSH).

Firstly, we determined the malondialdehyde (MDA) concentration to inspect the level of lipid peroxidation and then measured the levels of antioxidants. Earlier studies have suggested increased SOD activity in diabetic rats, which might be a protective measure against oxidative stress [49]. Our results show reduced SOD activity in animal groups that were treated with AG-Gnp. Moreover, CAT activity and GSH levels were also found to be increased in AG-Gnp-treated diabetic rats [50]. Reduced AMS activity is regarded as one of the factors showing impairment in the exocrine function of the pancreas [51]. In our experiment, AG-Gnp was more effective in improving the pancreatic exocrine function in comparison to AG alone [52]. Furthermore, the histopathological analysis provides evidence of improvement in pancreas tissue and viability of *β*-cell produced by AG-Gnp. Thus, we conclude that all the parameters discussed in this study implied the effectiveness of AG-Gnp in comparison to that of AG.

## 5. Conclusion

The present study provides an insight to understand the potential role of aminoguanidine-bioconjugated gold nanoparticles (AG-Gnp) in reducing diabetes-associated secondary complications by inhibiting glycation and generation of AGEs. The antiglycation property of AG-Gnp might provide a link that facilitates the glycobiologists to explore new therapeutic applications for halting the menace of AGEs. This novel approach increases the efficacy of aminoguanidine at reduced concentration with no toxicity and might effectively minimize the use of other antiglycation drugs that are toxic at high concentrations.

## 6. Future Prospects

Gold nanoparticles (Gnp) are among the most commonly used nanostructures in biological applications that are used in several therapeutic applications for the treatment of various diseases. Therefore, the bioconjugation of antiglycating agents such as aminoguanidine, pyridoxamine, and metformin with Gnp would help to reduce the cytotoxic concentrations of these drugs nowadays. Moreover, the bioconjugation of plant-derived natural products may also be done in order to get rid of the complications associated with the glycation reaction. Therefore, the need of the hour is to do the bioconjugation of natural products as well. This novel approach will be of prudent commercial value as no reports are there to inhibit the glycation reaction using bioconjugation of these drugs using Gnp. Moreover, the direct effect of Gnp on glycation status would also add reliable implications in its inhibition. The research on inhibition of glycation is at the nascent stage, and very few studies have been performed. The PubMed search itself shows the reality that subtle data is available in this regard till now. Therefore, the prospect of our investigation is reasonably alluring and might add more insights to stop the menace of glycation in the near future.

## Figures and Tables

**Table 1:** Effect of AG and AG-Gnp on total lactate dehydrogenase (LDH) released in the incubation medium from HSA-MGO mixtures.

Incubation time (min.)	Control	AG (10 mM)	AG-Gnp (0.5 mM)	AG-Gnp (1.0 mM)
0	0.00 ± 0.00	0.00 ± 0.00	0.00 ± 0.00	0.00 ± 0.00
30	0.58 ± 0.32	2.97 ± 0.43	1.27 ± 0.22	1.25 ± 0.27
60	1.28 ± 0.24	4.29 ± 0.43	2.33 ± 0.33	2.15 ± 0.24
90	1.46 ± 0.21	5.93 ± 0.14	3.35 ± 0.29	3.29 ± 0.26
120	2.65 ± 0.24	7.13 ± 0.23	3.91 ± 0.35	4.13 ± 0.41

Results are presented as mean ± SD.

**Table 2:** Effects of AG and AG-Gnp on blood glucose, triglycerides, and insulin in a diabetic rat model.

Group	Glucose (mmol L^−1^)	Triglycerides (mmol L^−1^)	Insulin (mmol L^−1^)
Control	3.67 ± 0.26	1.48 ± 0.23	9.87 ± 0.76
Diabetic	16.8 ± 1.13	13.56 ± 0.83	3.23 ± 0.42
Diabetic+AG (10 mM kg^−1^)	10.13 ± 0.48	7.64 ± 0.65	4.55 ± 0.29
Diabetic+AG-Gnp (0.5 mM kg^−1^)	8.67 ± 0.86	5.88 ± 0.53	5.32 ± 0.68
Diabetic+AG-Gnp (1.0 mM kg^−1^)	7.32 ± 0.76	4.51 ± 0.36	7.45 ± 0.47

Values are mean ± SD of 8 independent calculations.

**Table 3:** Effects of AG and AG-Gnp on antioxidant defense system activity and pancreatic exocrine function in a diabetic rat model.

Group	MDA (nmol L^−1^)	SOD (U mL^−1^)	CAT (U mL^−1^)	GSH (mg dL^−1^)	AMS (U dL^−1^)
Control	2.45 ± 0.68	140.2 ± 12.2	4.22 ± 1.87	172.9 ± 13.5	719.8 ± 23.7
Diabetic	4.23 ± 0.89	201.9 ± 21.7	1.48 ± 0.83	123.4 ± 12.5	617.3 ± 58.4
Diabetic+AG (10 mMg)	3.27 ± 0.98	136.3 ± 56.4	3.21 ± 1.14	155.8 ± 42.3	630.4 ± 93.5
Diabetic+AG-GNP (0.5 mM kg^−1^)	3.13 ± 0.43	130.2 ± 43.6	3.9 ± 1.45	167.8 ± 34.6	670.8 ± 72.6
Diabetic+AG-GNP (1 mM kg^−1^)	2.87 ± 0.42	136.6 ± 25.2	4.1 ± 1.11	178.1 ± 34.6	701.4 ± 68.5

AG: aminoguanidine; AG-Gnp: aminoguanidine gold nanoparticle; MDA: malondialdehyde; SOD: superoxide dismutase; CAT: catalase; GSH: reduced glutathione; AMS: amylase. Values are the mean ± SD of 8 independent calculations.

**Table 4:** Level of serum AGEs and CML-AGEs as measured by ELISA in diabetic and AG-Gnp-treated groups.

Group	Serum AGE level (*μ*g mL^−1^)	Serum CML level (ng mL^−1^)
Control	1.21 ± 0.23	0.52 ± 0.16
Diabetic	5.83 ± 0.45	2.12 ± 0.84
Diabetic+AG (10 mM)	4.92 ± 0.63	2.01 ± 0.23
Diabetic+AG-Gnp (0.5 mM kg^−1^)	4.14 ± 0.53	1.68 ± 0.33
Diabetic+Ag-Gnp (1.0 mM kg^−1^)	3.21 ± 0.71	1.23 ± 0.18

AG: aminoguanidine; AG-Gnp: aminoguanidine gold nanoparticle. Values are the mean ± SD of 3 independent calculations.

## Data Availability

The data for this article is within the manuscript.

## Abbreviations

AGEs: Advanced glycation end products
AG: Aminoguanidine
AG-Gnp: Aminoguanidine-gold nanoparticles
FL: *ε*-Fructosyl-L-lysine
GO: Glyoxal
Gnp: Gold nanoparticles
HPLC: High performance liquid chromatography
HSA: Human serum albumin
MGO: Methylglyoxal
CML: -*N*^*ε*^-(Carboxymethyl)lysine
CEL: -*N*^*ε*^-(Carboxyethyl)lysine
LDH: Lactate dehydrogenase
OPA: o-Phthalaldehyde
ROS: Reactive oxygen species
RCS: Reactive carbonyl species
RP-HPLC: Reverse phase-high performance liquid chromatography
3-DG: 3-Deoxyglucosone
DTNB: 5,5′-Dithio-bis-[2-nitrobenzoic acid]
EDC: 1-Ethyl-3-(3-dimethylaminopropyl)-carbodiimide.
